# Effect of Gold Nanostars Plus Amikacin against Carbapenem-Resistant *Klebsiella pneumoniae* Biofilms

**DOI:** 10.3390/biology11020162

**Published:** 2022-01-20

**Authors:** John Jairo Aguilera-Correa, Rafaela García-Álvarez, Aranzazu Mediero, Jaime Esteban, María Vallet-Regí

**Affiliations:** 1 Departamento de Química en Ciencias Farmacéuticas, Instituto de Investigación Sanitaria Hospital 12 de Octubre i+12, Universidad Complutense de Madrid, Plaza Ramón y Cajal s/n, 28040 Madrid, Spain; john_j2a@hotmail.com (J.J.A.-C.); rpgalvarez@ucm.es (R.G.-Á.); 2 Networking Research Centre on Infectious Diseases (CIBERINFEC), 28029 Madrid, Spain; 3 Bone and Joint Unit, IIS-Fundación Jiménez Diaz, UAM, Avenida Reyes Católicos, 2, 28037 Madrid, Spain; aranzazu.mediero@quironsalud.es; 4 Clinical Microbiology Department, IIS-Fundación Jiménez Diaz, UAM, Avenida Reyes Católicos, 2, 28037 Madrid, Spain; 5 Networking Research Centre on Bioengineering, Biomaterials and Nanomedicine (CIBER-BBN), 28029 Madrid, Spain

**Keywords:** carbapenem-resistant *K. pneumoniae*, gold nanostars, amikacin, biofilm

## Abstract

**Simple Summary:**

Carbapenem-resistant *Klebsiella pneumoniae* (CR-KP) infection rates represent a challenging treatment since the pipeline for effective antibiotics against this pathogen, such as beta-lactams among others, is practically nil. This study aims to evaluate the antibacterial effect of gold nanostars (GNS) alone or associated with some of the most widely used antibiotics for the treatment of CR-KP strains, i.e., meropenem or amikacin, on both planktonic or free-living and sessile forms. GNS were able to inhibit the planktonic growth of CR-KP at 80 µM, to eradicate the bacterial viability at 160 µM, and were unable to inhibit or eradicate the biofilm growth of this bacterium. GNS gave rise to filamentous bacteria through mechanisms mediated by the inhibition of energy-dependent cytoplasmic proteases. The combination of GNS and amikacin was able to inhibit or even eradicate the CR-KP biofilm. This combination was administered to greater wax moth larvae (*Galleria mellonella*), and this treatment was found to be tolerated well and to prevent the CR-KP infection. Thus, GNS in combination with amikacin represent a promising anti-CR-KP nanomaterial.

**Abstract:**

(1) Background: Carbapenem-resistant *Klesiella pneumoniae* (CR-KP) infection rates depict an almost pre-antibiotic scenario since the pipeline for effective antibiotics against this pathogen has been almost entirely depleted. This study aims to evaluate the antibacterial effect of gold nanostars (GNS) alone or associated with some of the most widely used antibiotics for the treatment of CR-KP strains, i.e., meropenem or amikacin, on both planktonic and sessile forms. Additionally, we measured the effect of GNS on cell proliferation and biocompatibility in invertebrate in vivo models. (2) Materials and methods: GNS were made from gold seeds grown using a seeded-growth surfactant-free method assisted by silver ions and functionalized with mercapto-poly(ethylene glycol)amino by ligand exchange. The antimicrobial capacity, effect on cell proliferation, and biocompatibility of the most effective combination was evaluated in a *Galleria mellonella* model. (3) Results: The minimum inhibitory concentration (MIC) and minimal bactericidal concentration (MBC) were 80 and 160 µM of GNS for all strains, respectively. The minimum biofilm inhibitory concentration (MBIC) and minimum biofilm eradication concentration (MBEC) were >320 µM of GNS for both. A synergy was found between GNS and amikacin. Larvae administered GNS plus amikacin were found to tolerate the treatment well, which prevented infection. (4) Conclusions: GNS are a promising anti-CR-KP nanomaterial.

## 1. Introduction

*Klebsiella pneumoniae* is a Gram-negative, non-motile, aerobic rod-shaped encapsulated bacterium. This species is considered an opportunistic pathogen capable of colonizing mucosal surfaces without causing infection. This bacterium is present in the stools of 5% to 38% and in the nasopharynx of 1% to 6% of individuals in the general community [1]. This microorganism can spread to other tissues, causing life-threatening infections. *K. pneumoniae* is the second leading cause of bloodstream infections caused by Gram-negative bacteria [2]. Bloodstream infections generally occur secondary to an infection of the urinary, gastrointestinal, or respiratory tract or following placement of intravenous or urinary catheters [2,3]. The rate of mortality related to *K. pneumoniae* bacteremia or pneumonia is higher than 20% [2,4]. Multi-drug-resistant *K. pneumoniae* is one of the most vexing issues in bacterial resistance [5]. Currently, carbapenem-resistant *K. pneumoniae* is one of the main bacterial pathogens associated with hospital outbreaks worldwide [6]. Most mechanisms of *K. pneumoniae* antibiotic resistance are acquired through horizontal gene transfer and mainly confer high-level resistance to antibiotics of the beta-lactam and quinolone types. Mechanisms of beta-lactam resistance are mediated by alterations of penicillin-binding proteins (PBPs), outer membrane permeability modifications (OmpK35 and OmpK36), active ejection pumps such as AcrAB and OqxAB pumps, and/or beta-lactamases capable of cleaving the beta-lactam ring inactivating these antibiotics, such as carbapenemases (e.g., KPC, NDM, VIM, OXA-48-like) [7,8]. Mechanisms of fluoroquinolone resistance are the result of point mutations in specific areas of DNA gyrase (genes *gyr*A and *gyr*B) and topoisomerase IV (genes *par*C and *par*E), acquired mechanisms such as Qnr, altered permeability, and/or efflux pumps [8]. Furthermore, this bacterial species can develop biofilm, a microbially derived sessile community characterized by cells that are irreversibly attached to a substratum or interface, are embedded in a self-produced matrix of extracellular polymeric substances and show an altered growth rate and gene transcription [9]. This form of growth can deactivate or repel antimicrobials, prevent host immune cell penetration, and block diffusion within it, among others [10].

The optimal treatment option for multi-drug-resistant *Klebsiella pneumoniae* infections is still not well established, but combination therapies including high-dose meropenem, aminoglycosides, colistin, fosfomycin, and tigecycline are widely used despite suboptimal results [11]. Among these combinations, carbapenems and aminoglycosides have shown a synergistic activity against carbapenem-resistant *K. pneumoniae* isolates [12] and has been found to be effective in vivo against pneumonia caused by this pathogen in a mouse model [13], and clinically, due to its low mortality rate [14].

Despite this, in the recent World Health Organization (WHO) report, *Global antimicrobial resistance and use surveillance system report: 2021* [15], carbapenem resistance rates in *K. pneumoniae* are described as an almost pre-antibiotic scenario since the pipeline for effective antibiotics against this bacterium has been diminished almost entirely [15]. For this reason, this bacterium is included in the list of WHO priority pathogens for research and development of new antibiotics [16]. This is where nanomedicine may have something to contribute. Nanomedicine is a multidisciplinary field focused on the application of nanotechnology for medical purposes and where nanomaterials are used for diagnosis, monitoring, prevention, and treatment of infectious and non-infectious diseases due to their ability to carry drugs or to act as a therapeutic agent [17,18,19,20,21,22,23,24,25]. Among these nanomaterials, gold nanostars (GNS) are a noteworthy example. GNS have a multi-branched structure and unique physicochemical properties [26] that make them promising tools for Biology and Medicine [27]. As a result, these nanomaterials have attracted significant attention in recent years due to their high biocompatibility and lack of toxicity [28]. Regarding their antibacterial applications, Feng et al. [29] have used GNS to synergistically kill a multi-resistant bacterium, methicillin-resistant *Staphylococcus aureus*, by using a two-dimensional reduced graphene-oxide-supported GNS nanocomposite, thanks to the intrinsic antimicrobial activity of GNS and the photothermal ablation made possible by GNS.

This study aims to evaluate the antibacterial effect of GNS alone or in association with meropenem or amikacin, two of the most widely used antibiotics for the treatment of three carbapenem-resistant *K. pneumoniae* strains on both planktonic and sessile forms. A possible mechanism of action by GNS is proposed based on the results of a synergy assay. Furthermore, we studied the effect of GNS on fibroblastic proliferation and biocompatibility in *Galleria mellonella* larvae in vivo models.

## 2. Materials and Methods

### 2.1. Nanostars

Hydrogen tetrachloroaurate trihydrate (HAuCl_4_·3H_2_O, ≥99.9%), trisodium citrate (≥98%), silver nitrate (AgNO_3_, ≥99.0%), hydrochloric acid (HCl, 37%), and l-ascorbic acid (AA, ≥99%) were purchased from Sigma Aldrich. Mercapto-poly(ethylene glycol) amino (α-Mercapto-ω-amino PEG hydrochloride, M_w_ 5 K) was purchased from Rapp Polymere. Milli-Q water was used in all experiments. All glassware was washed with *aqua regia*, rinsed with water, and dried prior to use.

Gold seeds of 14 nm in diameter were synthesized by the Turkevich method [30] based on the reduction of Au^3+^ to Au^0^ by using trisodium citrate under high temperatures. In short, an aqueous solution of HAuCl_4_ (0.5 mM, 250 mL) is placed on a hotplate at slow stirring and high temperature (100 °C). Once the boiling point was reached, trisodium citrate (47 mM, 12.5 mL) was rapidly added under vigorous stirring. The mixture was allowed to react for 15 min. The final solution had a gold concentration of 0.489 mM. GNS of different spike lengths were synthesized using a seeded-growth, surfactant-free method assisted by silver ions. An aqueous 250-mL solution containing HAuCl_4_ (1.25 mL, 50 mM) and HCl (0.25 mL, 1 M) was prepared, followed by fast addition of gold seeds (1.25 mL, 0.5 mM), AgNO_3_ (5 mL, 10 mM) and AA (1.25 mL, 100 mM) under vigorous stirring. The mixture was allowed to react for 30 s [31]. Lastly, GNS were functionalized with mercapto-poly (ethylene glycol) amino (Mw, 5 K) by ligand exchange. An aqueous solution of PEG (2 mg in 2 mL) was added dropwise to the as-synthesized GNS under vigorous stirring. The mixture was allowed to react for 1 h. Functionalized GNS were washed at least three times by centrifugation at different speeds depending on the size of the GNS and, finally, redispersed in milli-Q water. Time and speed of centrifugation were optimized previously: GNS were synthesized, functionalized and visualized by transmission electron microscopy (TEM) in order to determine their dimensions and thus select an adequate time and speed of centrifugation.

GNS were visualized by TEM. TEM images were obtained with a JEOL JEM 1400 transmission electron microscope operating at an acceleration voltage of 120 Kv using carbon-coated 400-square mesh copper grids. For visualization, samples were prepared by placing a drop of the previously diluted colloidal solution onto the copper grid. Excess of suspension was removed using filter paper. The GNS were characterized in terms of UV-visible optical extinction spectra, Dynamic Light Scattering (DLS), and ζ-potential. UV-visible optical extinction spectra of GNS were recorded using a Unicam UV-500 UV-visible spectrophotometer by diluting GNS in milli-Q water. LDS and ζ-potential measurements were recorded using a Zetasizer Nano ZS (Malver Pananylitical, Grovewood Road, Malvern, UK). For size distribution measurements, samples were diluted with milli-Q water in cuvettes of 1-cm optical step. Each sample was measured in triplicate.

### 2.2. Microbiological Studies

Two different carbapenem-resistant *K. pneumoniae* strains were chosen for this study: a collection strain from American Type Culture Collection (ATCC), ATCC 23357, and a clinical strain isolated in the Microbiology Department of the Fundación Jiménez Díaz University Hospital from a 76-year-old man with a urinary tract infection (KP52) and a 76-year-old woman, also with a urinary tract infection (KP5). All strains were kept frozen at −80 °C until experiments were performed. The antibiogram of each strain was performed by using VITEK^®^ 2 automated system (Biomérieux, Marcy-l’Étoile, France) and the type of carbapenemase was determined by using Unyvero i60^®^ multiplex PCR (Curetis AG, Holzgerlingen, Germany).

#### 2.2.1. Minimum Inhibitory Concentration and Minimum Bactericidal Concentration

Minimum inhibitory concentrations (MICs) were determined using the previously described broth microdilution method [32]. In brief, a series of two-fold dilutions of GNS in concentration from 320 to 0.3125 µM were added to cation-adjusted Müeller–Hinton broth (Sigma Aldrich, St. Louis, MO, USA) to a final volume of 100 μL per well to a Costar 96-well round-bottom polypropylene plate (Corning Inc., Corning, NY, USA). Thereafter, 100 µL of bacterial suspension in cation-adjusted Müeller–Hinton broth (CAMHB) containing approximately 10^5^ colony-forming units per milliliter (CFU/mL) was added to each well and the plate was statically incubated at 37 °C and 5% CO_2_. After incubation, MIC was determined using a modification of the methodology, previously described by other authors [33]. For this, 10 μL of alamarBlue^®^ (BIO-RAD, Hercules, CA, USA) [34] were added per well and the plate was incubated at 37 °C and 80 rpm for 1 h [35]. On completion of the incubation, columns with no color change were scored as above the MIC value and the fluorescence was measured using an excitation wavelength of 560 nm and emission wavelength of 590 nm. Minimum bactericidal concentrations (MBCs) were determined using the flash microbicide method, previously described [36]. The MBC is defined as the minimum concentration required to kill a certain bacterial concentration. Briefly, 20 μL of each well after 24-h incubation were mixed with 180 μL of tryptic soy broth in a new 96-well plate, which incubated statically at 37 °C and 5% CO_2_ for 24 h. After incubation, MBC was determined measuring absorbance using a wavelength of 600 nm. These experiments were performed four times.

To visually support the numerical results, the previous experiment was analyzed using TEM. The protocol for TEM has been described previously [37]. Semithin sections (0.6 μm) for light microscopy and thin sections (60 nm) for TEM of resin-included bacteria were cut using a Leica Ultracut ultramicrotome UC7 (Leica Wetzlar, Germany). Sections were collected on 200 mesh nickel grids and examined using a Jeol JEM 1400 transmission electron microscope (Jeol Ltd., Tokyo, Japan).

#### 2.2.2. Minimum Biofilm Inhibitory Concentration and Minimum Biofilm Eradication Concentration

Minimum biofilm inhibitory concentrations (MBICs) and minimum biofilm eradication concentrations (MBECs) were determined using the methodology previously described [38]. The MBIC is the minimum concentration required to inhibit the visible growth of a bacterial biofilm. For MBIC, biofilm formation on the bottom of a MicroWell 96-well flat-bottom plate (Thermo Fisher Scientific, Waltham, MA, USA) was induced by inoculating 100 µL of CAMHB containing 10^5^ CFU/mL of bacteria per well and the plate was incubated at 37 °C and 5% CO_2_ for 24 h [38]. After incubation, the supernatant was aspirated, 200 µL per well with the different concentrations was deposited and the plate was incubated at 37 °C and 5% CO_2_ for at least 20 h. After incubation, MBIC was determined by using alamarBlue^®^, as described above, and measuring fluorescence. The MBEC is the minimum concentration required to kill a bacterial biofilm. For MBEC, the biofilm grown on the bottom of each well was scraped and mixed with the supernatant. Thereafter, 20 μL of each well was mixed with 180 μL of tryptic soy broth in a new 96-well plate, which incubated statically at 37 °C and 5% CO_2_ for 24 h. After incubation, MBEC was determined by measuring absorbance using a wavelength of 600 nm. These experiments were performed four times.

#### 2.2.3. Synergy between Amikacin or Meropenem and GNS against Carbapenem-Resistant *K. pneumoniae* Biofilm

Synergy between amikacin or meropenem and GNS against *K. pneumoniae* biofilm was studied by using a modification of the methodology previously described [39,40]. Briefly, *K. pneumonia* biofilm was grown in CAMHB on a flat-bottom 96-well plate at 37 °C and 5% CO_2_ for 24 h, as mentioned previously. Then, the supernatant of each well was aspirated and 200 µL of CAMHB with the corresponding concentration of GNS and a constant concentration of antibiotic was deposited onto the biofilm grown. The constant concentration of amikacin (4 µg/mL) and meropenem (2 µg/mL) was selected because the biofilm of both strains showed resistance to them. One well with only CAMHB was used as a positive control. After 24 h of incubation at 37 °C and 5% CO_2_, MBIC and MBEC were determined using the above-mentioned methodology. This experiment was performed four times.

To visually support the numerical results, the previous experiment was analyzed using scanning electron microscopy (SEM). Biofilms were grown on Whatman^®^ polycarbonate membrane (Merck, Darmstadt, Germany) and fixed with 2.5% glutaraldehyde in 0.1 M sodium cacodylate buffer at pH 7 at 4 °C for 90 min. Samples were then dehydrated with increasing concentrations of ethanol (30, 50, 70, 90, and 100%) at 22 °C for ten min. Micrographs were obtained using a field emission gun JEOL JSM6400 scanning electron microscope (Jeol Ltd., Tokyo, Japan).

#### 2.2.4. Antibacterial Mechanism of GNS

To determine one of the possible antibacterial mechanisms of GNS against carbapenem-resistant *K. pneumoniae*, only the KP52 strain was used. The interaction type between aztreonam (AT) or imipenem (IP) and GNS against carbapenem-resistant *K. pneumoniae* in planktonic form was studied by using the checkerboard [39,40]. This methodology is supported by the European Committee on Antimicrobial Susceptibility Testing (EUCAST) [41]. The interaction between each antibiotic and GNS can be quantified by the fractional inhibitory concentration (FIC). For two antibacterial compounds, A and B, acting individually or in combination:

$$\mathrm{FIC}\;\mathrm{Index}=\frac{A}{{\mathrm{MIC}}_{A}}+\frac{B}{{\mathrm{MIC}}_{B}}$$

where A is the MIC of AT or IP and B is the MIC of GNS in combination, and MIC_A_ and MIC_B_ are the MIC of AT or IP and GNS individually. The FIC index is the sum of FIC_A_ and FIC_B_. An FIC index of <0.5 points out synergism, >0.5–1 additive effects, >1 but <4 indifference, and ≥4 is considered to be antagonism [42].

An Ε-test^®^ susceptibility study (Biomeriéux, France) was performed to estimate the subinhibitory concentration of AZ and IP for KP52. After incubation, bacterial viability was determined by addition of 20 µL of 5 mg/mL of 3-(4,5-dimethylthiazol-2-yl)-2,5-diphenyltetrazolium bromide (MTT) (Sigma Aldrich, Merck, Darmstadt, Germany) [43] and incubating for 1 h at 37 °C and 5% CO_2_. This experiment was performed by duplicate and the mean was represented.

### 2.3. Cell Proliferation

The fHDF/TER166 fibroblast cell line (Evercyte, Vienna, Austria) were seeded in a concentration of 10,000 cells/cm^2^ on 96-well plates in DMEM with 10% fetal bovine serum and 1% penicillin-streptomycin (DMEM, Invitrogen, Thermo Fisher Scientific Inc., Waltham, MA, USA). Then, the cells were incubated at 37 °C and 5% CO_2_ overnight. Thereafter, the supernatant was removed and cells were incubated in the presence of serial dilutions of GNS from 320 to 10 µM for 48 h (n = 8 per concentration). After incubation, cell proliferation was determined by adding 20 µL of 5 mg/mL of MTT [43] and incubating for 3 h at 37 °C and 5% CO_2_. This experiment was performed in triplicate.

### 2.4. Galleria Mellonella Model

We stored larvae of *G. mellonella* (Harkito Reptile, Madrid, Spain) at room temperature and in darkness before use. The carbapenemase-producing *K. pneumoniae* strain used in this model was KP5. This strain was chosen over others because of its specific ability to proliferate and cause the death of *Galleria mellonella* larvae. We infected groups of 10 larvae with 10 μL of bacterial suspension of KP5 (1.00 ± 0.02 McFarland turbidity scale, approximately 10^8^ colony-forming units per milliliter) by intrahemocoelic injection through the last left proleg and incubated at 37 °C. Larvae survival was evaluated at 24-h intervals for one week. A group inoculated with sterile saline (saline) and non-manipulated groups (control) were used as negative controls. The anti-*K. pneumoniae* ability of GNS plus amikacin was demonstrated by injecting 10 μL of 4 µg/mL of amikacin plus 160 µM of GNS in saline after 1 h of *K. pneumoniae* inoculation. We recovered the inoculated *K. pneumoniae* strain from each dead larva by means of culture in Brilliance Carbapenem-resistant enterobacteria CRE agar (Oxoid, Thermo Fisher Scientific Inc., Waltham, MA, USA).

### 2.5. Statistical Analysis

We performed statistical analysis by using Stata Statistical software, Release 11 (StataCorp2009). We used the non-parametric Wilcoxon test for a pairwise comparison using median and interquartile range values. For the *G. mellonella* larvae model, we used a log-rank test to perform a pairwise comparison of the Kaplan–Meier survival curves of two groups. We considered *p*-values < 0.05 to denote statistical significance in all tests.

## 3. Results

### 3.1. GNS Characterization

**Figure 1:**
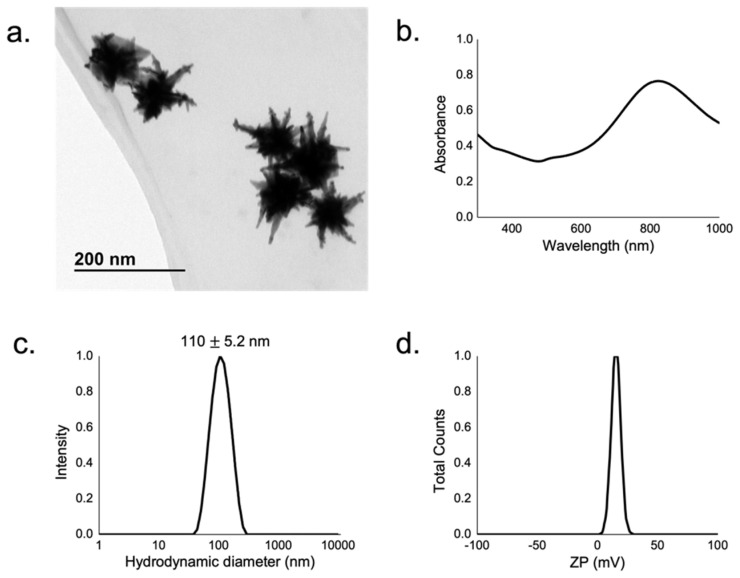
Physico-chemical characterization of gold nanostars (GNS). TEM image (**a**), UV-visible optical extinction spectra at 0.13 mM (**b**), DLS (**c**), and ζ-potential of GNS (**d**).

The synthesis of GNS was performed by a seeded-growth method reported in the literature by Yuan et al. [31] in which the authors avoid the use of a surfactant, thereby facilitating the functionalization step. As-synthesized GNS were then functionalized with thiolated PEG (Mw = 5000 kDa) with amino groups in order to stabilize the GNS and confer a positive surface charge. The characterization of GNS is summarized in Figure 1, where TEM images (Figure 1a), UV-visible spectra (Figure 1b), DLS (Figure 1c), and zeta-potential measurements of GNS can be observed (Figure 1d).

TEM images revealed well-dispersed nanoparticles with their expected star shape and a diameter of 103.8 ± 11.0 nm. The tip-confined localized surface plasmon resonance LSPR of these nanoparticles was 810 nm, as determined by UV-visible spectroscopy. Colloidal stability was analyzed by DLS measurements, which revealed a hydrodynamic diameter of 110.0 ± 5.2 nm and gave no signs of aggregation. Finally, zeta-potential measurements of these GNS yielded a value of +14.0 ± 0.4 mV, which can be explained by the functionalization with an amino-terminated PEG.

### 3.2. Microbiological Studies

The antibiotype of the *K. pneumoniae* strains used is shown in Table 1. An OXA-48 carbapenemase was detected in KP52, while an OXA-48 carbapenemase, a CTX-M broad-spectrum β-lactamase and aminoglycoside 6′-N-acetyltransferase type I were detected in KP5 by Unyvero i60^®^ multiplex PCR.

#### 3.2.1. Minimum Inhibitory Concentration (MIC), Minimum Bactericidal Concentration (MBC), Minimum Biofilm Inhibitory Concentration (MBIC), and Minimum Biofilm Eradication Concentration (MBEC)

The antibacterial effect of the nanoparticles was evaluated by studying the MIC and MBC. For this purpose, two planktonic carbapenem-resistant *K. pneumoniae* strains were exposed to different concentrations of GNS. The MIC and MBC were found to be 80 and 160 µM for all strains.

**Figure 2:**
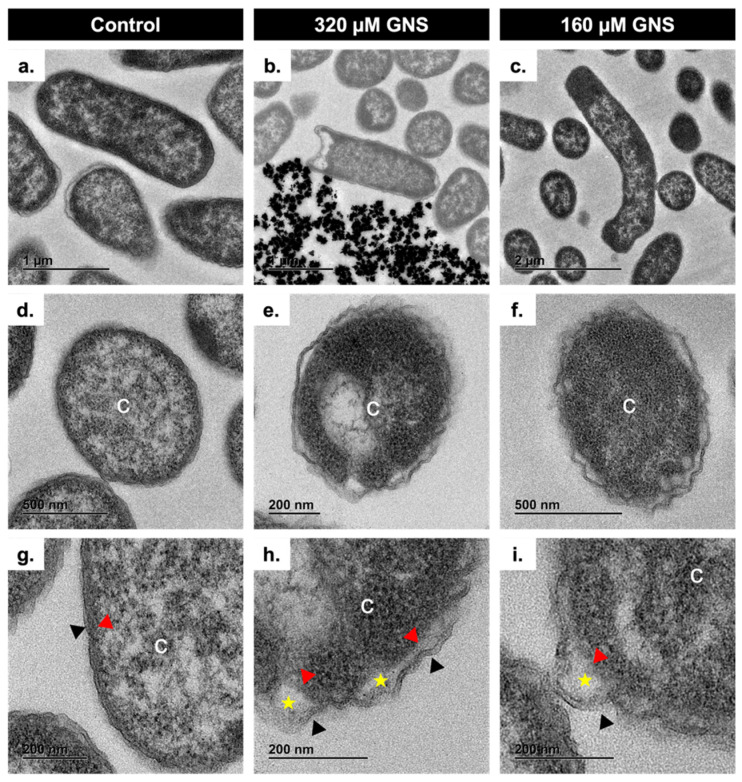
TEM images of KP52 faced against 0 (control) (**a**,**d**,**g**), 320 (**b**,**e**,**h**), and 160 µM(**c**,**f**,**i**) of gold nanostars (GNS). C: cytoplasm. Black triangle: outer membrane border. Red triangle: cytoplasmatic membrane. Stars: periplasmatic vacuoles.

To visually support the numerical results, the antibacterial effect of the GNS was analyzed for KP52 at 0 (control), 320, and 160 µM in CAMHB (Figure 2). Bacteria from the control condition showed a anodyne appearance (Figure 2a,d,g), with a cytoplasmatic membrane so intimately bound to the outer membrane that the periplasmic space was negligible (Figure 2d,g). Some bacteria faced against 320 µM of GNS showed important outer membrane damages able to seriously compromise bacterial viability (Figure 2b), while some bacteria faced against 160 µM of GNS showed short filamentous cells consisting of at least two bacteria (Figure 2c). Furthermore, most of the bacteria faced against 320 µM of GNS (Figure 2e,h) and some bacteria faced against 160 µM of GNS (Figure 2f,i) showed a wavy outer membrane and periplasmatic vacuoles that separated the outer membrane from the cytoplasmatic membrane without altering the integrity of the latter.

The antibiofilm effect of GNS was evaluated by studying the MBIC and MBEC. To do this, different GNS were faced against the biofilm of the two carbapenem-resistant *K. pneumoniae* strains at different concentrations of GNS. The MBIC and MBEC of GNS against the biofilm of the two carbapenem-resistant *K. pneumoniae* strains were found to be >320 µM for both MBIC and MBEC.

#### 3.2.2. Synergy between Amikacin or Meropenem Plus GNS against Carbapenem-Resistant *K. pneumoniae* Biofilm

**Figure 3:**
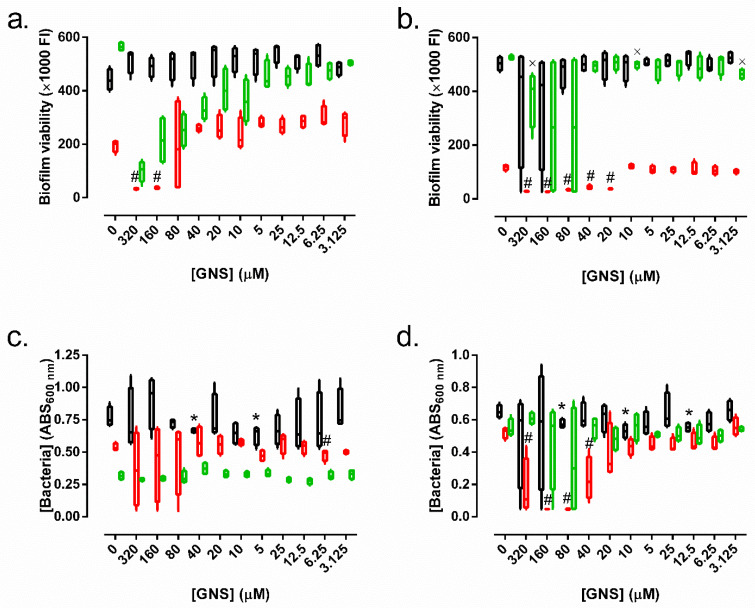
Minimum biofilm inhibitory concentrations (**a**,**b**) and minimum eradication biofilm concentrations (**c**,**d**) of *K. pnemoniae* ATCC23357 (**a**,**c**) and carbapenem-resistant KP52 (**b**,**d**) biofilms faced against different concentrations of gold nanostars (GNS) (black) and with 4 µg/mL of amikacin (red) or with 2 µg/mL meropenem (green). Boxes and bars represent the median and interquartile range and 10th and 90th percentiles, respectively. Note: At [GNS] = 0 μM, the bars represent the biofilm viability or bacterial concentration of the *K. pneumoniae* strain without GNS (black), and in presence of 4 mg/L of amikacin (red) or 2 mg/L pf meropenem (green). *: *p*-value < 0.05 for Wilcoxon test between control (untreated) biofilm and biofilms treated with different concentrations of GNS. #: *p*-value < 0.05 for Wilcoxon test between biofilm treated only with amikacin and biofilms treated with different concentrations of GNS plus amikacin. ×: *p*-value < 0.05 for Wilcoxon test between biofilm treated only with meropenem and biofilms treated with different concentrations of GNS plus meropenem.

Results of the synergy between amikacin plus GNS and meropenem plus GNS against ATCC23357 and KP52 biofilm are represented in Figure 3. Only the combination of 4 µg/mL of amikacin plus concentrations of GNS higher than 80 µM of GNS were able to inhibit the biofilm growth of both *K. pneumoniae* strains (Figure 3a,b). This combination eradicated the KP52 biofilm at 80 and 160 µM of GNS (Figure 3d), unlike the ATCC 23,357 biofilm, which was resistant to concentrations higher than 320 µM of GNS (Figure 3c).

On the other hand, the combination of 2 µg/mL of meropenem plus different concentrations of GNS showed a GNS concentration-dependent inhibitory capacity on ATCC23357 biofilm (Figure 3a), but not on KP52 biofilm (Figure 3b). However, this combination was unable to eradicate both the ATCC23357 biofilm (Figure 3c) and KP52 biofilm (Figure 3d).

**Figure 4:**
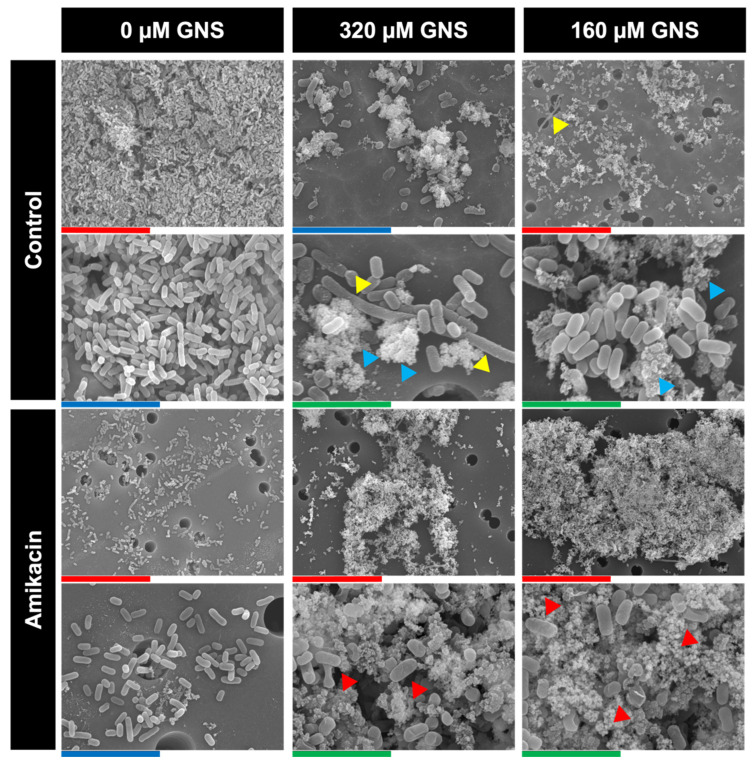
KP2 biofilm treated with 4 µg/mL of amikacin and two concentrations of GNS. Red, blue, and green bars represent 30, 10, and 5 µm, respectively. Yellow triangles denote filamentous cells. Blue triangles indicate GNS clusters. Red triangles signal bacteria with important structural damages.

The appearance of KP52 biofilm treated with the combination of amikacin plus 320 and 160 µM of GNS is shown in Figure 4. Untreated biofilm showed a normal appearance, a conglomerate of bacilli without structural damages. Biofilms treated with both 320 and 160 µM of GNS showed bacilli without structural abnormalities, GNS clusters inside (Figure 4, blue triangles) and filamentous cells of approximately 20 µm (Figure 4, yellow triangles). Bacteria from biofilm treated with amikacin only showed no structural alteration that could suggest a viability reduction. On the contrary, biofilms treated with amikacin and GNS did show bacteria with structural damages (Figure 4, red triangles), which pointed to a viability decrease.

This experiment was not performed with KP5 due to the presence of the aminoglycoside N-(6′)-acetyltransferase type 1 in this strain.

#### 3.2.3. Antibacterial Mechanism of GNS

**Figure 5:**
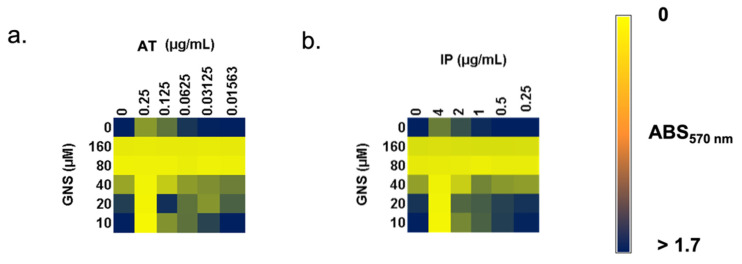
Synergy between gold nanostars (GNS) and aztreonam (AT) (**a**) or imipenem (IP) (**b**) against planktonic state of KP52.

The MICs of KP52 for AT and IP were found to be 0.38 and 8 µg/mL, respectively. The subinhibitory concentrations of each antibiotic, 0.25 and 4 µg/mL, respectively, were used for the synergy assay by using GNS, the results of which are represented in Figure 5. The FIC Index for AT and GNS was 1.66, while for IP and GNS it was 1.5. Both AT (Figure 5a) and IP (Figure 5b) showed indifference with GNS.

### 3.3. Cell Proliferation

**Figure 6:**
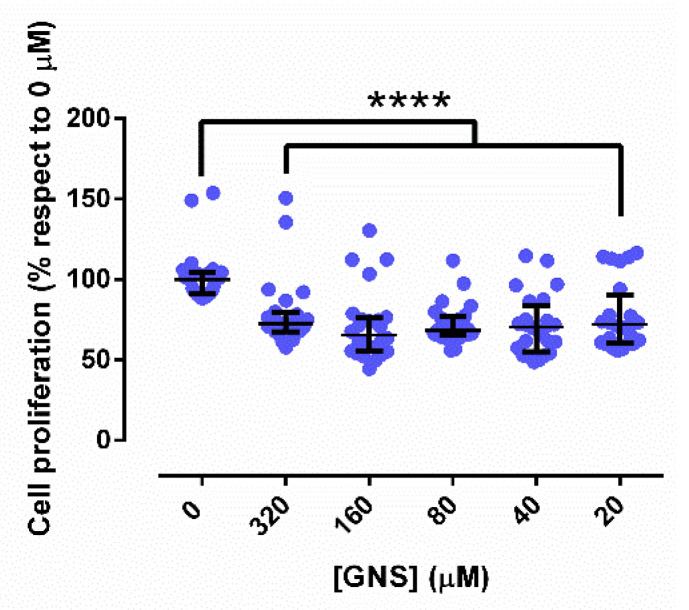
Fibroblastic proliferation in presence of different concentrations of gold nanostars (GNS). ****: *p*-value < 0.0001.

GNS showed a concentration-independent effect of fHDF/TER166 fibroblasts (Figure 6). GNS significantly decreased cell proliferation until 70.7 (61.8–78.1)%, irrespective of the concentration tested.

### 3.4. Galleria Mellonella Larvae Model

**Figure 7:**
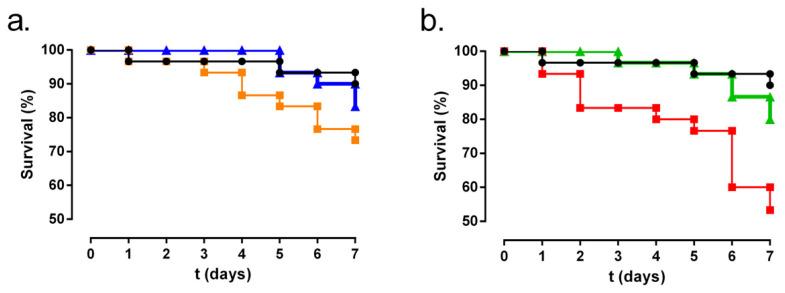
Survival of the greater wax moth (*G. mellonella*) over a week. (**a**) Biocompatibility of GNS plus amikacin (GNS+AMK). Black, orange, and blue lines represent the non-manipulated, saline, and GNS + AK groups, respectively. (**b**) Antibacterial effectiveness of GNS + AMK against carbapenem-resistant *K. pneumoniae* strain (KP5). Red and green lines represent KP5-infected and GNS + AMK-treated KP5-infected groups, respectively.

No differences in survival between the saline group, non-manipulated group, and GNS plus amikacin group were found (*p*-value = 0.100 for the saline and non-manipulated group, *p*-value = 0.479 for the saline and GNS plus amikacin group, and *p*-value = 0.303 for the non-manipulated and GNS plus amikacin group) (Figure 7a).

Statistically significant differences were observed when we compared survival between the KP5-infected group and KP5-infected group treated with GNS plus amikacin (*p*-value = 0.027) (Figure 7b). No differences in survival were found between the KP5-infected group treated with the GNS plus amikacin and non-manipulated group (*p*-value = 0.302) (Figure 7b).

## 4. Discussion

In this study, we demonstrate the inhibitory and bactericidal effect of GNS against *K. pneumoniae* with or without carbapenem resistance, showing a reduced effect on cell proliferation of fibroblasts in vitro. Furthermore, GNS plus amikacin showed an antibiofilm effect on the carbapenem-resistant strain, good biotolerance, and an anti-infective ability against a carbapenem-resistant strain by using *Galleria mellonella* in vivo model.

Our results reveal that GNS have an important bactericidal effect against *K. pnemoniae*. This antibacterial capacity stems from the antibacterial effect of gold released from the nanostars that show a high affinity for thiol groups from any bacterial protein [44]. The union between gold atoms and thiol groups provokes important modifications in the protein structure from membrane systems (outer or cytoplasmatic membrane) that comprise the membrane integrity and bacterial viability. Furthermore, gold can also cause an imbalance in membrane potential, inhibiting ATPase activity and protein synthesis by binding tRNA [45]. This antibacterial action of gold did not include reactive oxygen species (ROS)-related mechanism [45], but the ROS generation by gold nanoparticles during plasmonic photothermal therapy has been recently described [29,46]. Our results are consistent with those previously described by other authors, who described the antibacterial capacity of gold nanomaterials against *Staphylococcus aureus*, *Pseudomonas aeruginosa*, *Escherichia coli* [47], *Cutibacterium acnes* [48], and *Streptococcus pneumoniae* [49], among others.

One of the most important findings of this study is the ability of GNS to induce the formation of filamentous *K. pneumoniae* cells. PBPs are important proteins that are responsible for the construction of peptidoglycan, the major component of bacterial cell walls and can catalyze the glycan strand (transglycosylation) and cross-linking between glycan chains (transpeptidation) [50]. The blocking effect of GNS may be responsible for the appearance of periplasmic vacuoles between the outer membrane and the cytoplasmic membrane of *K. pneumoniae* observed by TEM (Figure 2e,h,f,i), but also the formation of filamentous cells observed by both TEM (Figure 2c) and SEM (Figure 4, yellow triangles). Filamentous cells are long, multi-nucleoid, and form when normal cells elongate and replicate their DNA but are unable to septate and divide [51,52]. These bacterial cells act as “normal” bacteria, as they can express genes normally and continue to synthesize flagella [52]. To corroborate this hypothesis, a synergy assay between PBP-specific antibiotics and GNS was performed. Aztreonam is a monobactam that shows a very high affinity for PBP3 and a moderate affinity for PBP1a [53]. Imipenem is a carbapenem that was shown to have very high binding affinities to PBP-2 and PBP-4 [54]. The use of these beta-lactams is due to their ability to induce the formation of filamentous bacteria *per se* [55,56]. The results obtained point out that the presence of filamentous cells does not reveal higher affinity of GNS for PBP3 or PBP1a, whose inactivation gives rise to filamentous cells [57,58]. The synergy assay revealed the lack of interaction between the two antibiotics tested and GNS, whereby GNS may give rise to filamentous bacteria through PBP-independent mechanisms. One of these mechanisms may be the inhibition of energy-dependent cytoplasmic proteases (e.g., ClpXP, Lon, and HslUV) [59], which would lead to the accumulation of SulA, a cell division inhibitor and SOS-regulated protein, whose accumulation inhibits cell division in *E. coli* [60].

*K. pneumoniae* is a bacterial species that is capable of biofilm development [61]; however, the antibacterial effect of GNS was not observed against *K. pneumoniae* biofilm. These findings are in agreement with the well-known resistance of bacterial biofilms to heavy metals, but contrast with descriptions of other authors, who have stated that gold nanoparticles had a strong antibiofilm effect on the biofilm of *S. aureus* [62] and *P. aeruginosa* [62,63]. Despite these results, when GNS are combined with one of the two most widely used antibiotics to treat carbapenem-resistant *K. pneumoniae* strains, amikacin or meropenem, both of those combinations inhibited *K. pneumoniae* biofilm growth (Figure 3a,b), but only the combination of GNS and amikacin eradicated the biofilm of carbapenem-resistant *K. pneumoniae* strain (Figure 3d). This observation would support the combination of GNS and amikacin as a promising treatment for infections caused by these strains where biofilm development is involved. This synergy between GNS and antimicrobial molecules has been scarcely described in the literature. Although there are some studies that reveal the synergy between metals and antibiotics [64,65,66], only Mu et al. [67], who used chitosan-streptomycin conjugates gold nanoparticles, observed that gold nanoparticles were responsible for facilitating the entry of streptomycin into *P. aeruginosa* biofilm. Therefore, GNS likely facilitates the entry of amikacin or imipenem into *K. pneumoniae* biofilm.

The effect of GNS on fibroblast proliferation was concentration-independent and only reduced the cell proliferation by 30% (Figure 6). The mechanism of inhibition of cell proliferation by gold nanomaterials is still unclear. However, some authors assert that gold can generate ROS, cause morphological change in cells as well as cytoskeleton defects, lead to cell damage and inhibition of proliferation, and interfere in the expression level of some proliferation-related genes [68]. Based on our results, GNS appears not to be fully biocompatible. Considering this effect on cell proliferation, the biotolerance of GNS was studied by using an *Galleria mellonella* model.

Recently, a *G. mellonella* larvae model has been proposed as an efficient pre-screening animal model to reduce the number of toxicity tests in murine models [69]. In our study, the combination of GNS and amikacin used, which is the most effective against *K. pneumoniae* in both planktonic and biofilm form, was found to be very well tolerated, as it was associated with similar mortality in controls (Figure 7a). These results agree with those reported by Moya-Andérico et al. [69], who showed the high tolerance of *G. mellonella* larvae to this type of nanoparticles, with a median lethal dose at 48 h of 2.023 mg of gold nanoparticles functionalized with containing histidine, arginine, and lysine amino acids per kilogram of larvae. Moreover, this combination was able to prevent infection of a carbapenem-resistant *K. pneumoniae* strain (Figure 7b). These results would support this combination as a possible and effective therapeutic alternative to other antibiotic combinations.

GNS in combination with amikacin is a potential new treatment against carbapenem-resistant *K. pneumoniae* strains, joining many other promising new nanoparticle-based strategies to fight this kind of infections, such as silver nanoparticles [70], imipenem-loaded poly-ε-caprolactone and polylactide-co-glycolide nanocapsules [71], or bovine serum albumin nanoparticles reinforced *K. pneumoniae* outer-membrane vesicles [72]; peptides, e.g., Komodo-dragon cathelicidin-inspired peptides [73]; or based on polycationic oligoethyleneimine [74].

This study is not exempt from limitations. First, the number of carbapenem-resistant strains used in this study is very limited. More studies need to be performed to corroborate these findings. Second, though GNS were biocompatible in the *G. mellonella* larvae model, a 30% reduction in fibroblast proliferation may indicate that they are not fully biocompatible. Hence, an in vivo murine study is necessary to corroborate the results obtained in the *G. mellonella* model.

## 5. Conclusions

GNS were found to be a promising nanomaterial against carbapenem-resistant *K. pneumoniae* strains. GNS were able to inhibit and kill the planktonic form of *K. pneumoniae*. Though GNS failed to inhibit the *K. pneumoniae* biofilm growth, the combination of GNS and amikacin or imipenem did inhibit growth of these biofilms, and only the combination of GNS and amikacin eradicated a carbapenem-resistant *K. pneumoniae* biofilm. Though GNS significantly reduced fibroblastic proliferation, they were biocompatible and able to prevent the carbapenem-resistant *K. pneumoniae* strain infection in *G. mellonella* larvae.

## Figures and Tables

**Table 1:** Antibiogram of *K. pneumoniae* strains used in this study. MIC: minimum inhibitory concentration (µg/mL). *: MIC determined by E-test^®^. BSBL: Broad-spectrum β-lactamase. S: susceptible. R: Resistant. I: intermediate.

Antibiotic	ATCC23357 (MIC)	KP52 (MIC)	KP5 (MIC)
Amikacin *	S (4)	S (2)	S (4)
Ampicilin	R (<32)	R (>32)	R (>32)
Cefotaxime	S (<0.25)	I (2)	R (64)
Cefoxitine	S (<4)	S (8)	S (8)
Ceftazidime	S (0.5)	S (0.5)	R (>64)
Gentamycin	S (<1)	S (<1)	S (<1)
Tobramycin	S (<1)	R (8)	R (8)
Imipenem	S (<0.25)	I (8)	R (>16)
Cefuroxime	S (8)	R (32)	R (>64)
Amoxicilin-clavulanic acid	S (8)	R (>32)	R (>32)
Ciprofloxacin	S (<0.25)	R (>4)	R (>4)
Fosfomycin	R (64)	R (>256)	R (>256)
Co-trimoxazole	S (<20)	R (>320)	R (>320)
Cefuroxime-axetil	S (8)	R (32)	R (>64)
Cefepime	S (<0.12)	S (0.5)	R (>32)
Colistin *	S (0.125)	S (0.019)	S (0.125)
Ertapenem	S (<0.12)	R (>8)	R (>8)
BSBL	Negative	Negative	Positive
Ceftazidime/avibactam	S (0.75)	S (0.5)	S (0.75)
Nadilixic acid	S (4)	R (>32)	R (>32)
Nitrofurantoin	S (<16)	R (128)	R (128)

## Data Availability

The data presented in this study are available on request from the corresponding author.
